# 3D food printing improves color profile and structural properties of the derived novel whole-grain sourdough and malt biscuits

**DOI:** 10.1038/s41598-022-16659-5

**Published:** 2022-07-19

**Authors:** Yusuf Olamide Kewuyemi, Hema Kesa, Reinout Meijboom, Oyekunle Azeez Alimi, Oluwafemi Ayodeji Adebo

**Affiliations:** 1 School of Tourism and Hospitality, College of Business and Economics, University of Johannesburg, Bunting Road Campus, P.O. Box 524, Gauteng, South Africa; 2 Department of Biotechnology and Food Technology, University of Johannesburg, Doornfontein Campus, P.O. Box 17011, Johannesburg, 2028 South Africa; 3 Department of Chemical Sciences, Research Centre for Synthesis and Catalysis, University of Johannesburg, Kingsway Campus, Auckland Park, P.O. Box 524, Johannesburg, 2006 South Africa

**Keywords:** Structure determination, Biotechnology

## Abstract

Presentation of foods is essential to promote the acceptance of diversified and novel products. This study examined the color profile, browning index (BI), and structural properties of 3D-printed and traditional biscuits from whole-grain (WG) sourdough and germinated flours. The processed flours and composite/multigrain flours comprising cowpea sourdough (CS) and quinoa malt (QM) were used to prepare the snacks, and their structural characteristics were determined. Compared with the traditional biscuits, the 3D-printed biscuits showed considerable distinction in terms of consistent structural design and color intensities. The in-barrel shearing effect on dough biopolymers, automated printing of replicated dough strands in layers, and expansion during baking might have caused the biscuits’ structural differences. The composite biscuit formulations had a proportional share of CS and QM characteristics. The 80% CS and 20% QM printed biscuit had a low redness and BI, increased cell volume, average cell area, and total concavity. The 60% CS and 40% QM printed snack showed improved lightness and yellowness, increased average cell elongation, and less hardness. The 3D-printed composite biscuits may be recommended based on their unique structural characteristics. Such attributes can enhance the acceptability of printed foods and reinvent locally prepared meals as trendy, sustainable, and functional foods.

## Introduction

Three-dimensional (3D) printing is one of the fourth industrial revolution techniques that can expand and reinvent foods with improved structure, appealing characteristics and facilitate personalized nutrition^1–3^. The most widely reported 3D food printing technique is the use of extrusion mechanisms^2,4^. In this processing technique, edible products are printed by the stepwise addition of food materials in layers to replicate a computer-aided design^5^. Its technical flexibility approach enables complex modulation of resultant structures with unique and better geometry than the available traditional products prepared by manual molding^6^. Derossi et al.^6^ highlighted that limited studies had investigated the impacts of 3D food printing per se (independently from printing variables) on the structural properties of the derived printed products. The authors conducted a study in that regard by comparing 3D-printed and hand-made rice and wheat-based formulations^6^. They reported that the 3D printing process intrinsically led to the creation of bigger pores but less in number and like-round in shape, which caused high chewiness, cohesiveness, and hardness of the obtained printed snacks. Further studies on similar novel products would broaden the understanding of the effect of 3D food printing on desirable and innovative features of the resulting products.

Recent literature has highlighted that the current research direction in 3D food printing applications includes the need to develop novel 3D-printed foods containing functional and bioactive constituents in addition to basic nutrients for wellness benefits^2,7,8^. Such new 3D-printed products are also expected to meet the attractive and acceptability demands of health-conscious consumers^9^. Several research studies have explored extrusion-based 3D food printing to develop novel 3D printed products^1,10^. However, Kewuyemi et al.^2^ specifically noted that there is limited data on the use of fermented and malted products (biomodified food ink) for 3D food printing. Besides, fewer investigations on 3D food printing applications had explored pretreated or biomodified food substrates as food ink^11^.

To attain personalized nutrition, less expensive pretreatment techniques such as fermentation and malting are vital to enhancing available nutritional and health-promoting components in food substrates before the 3D printing process^2^. The structural characteristics of such food matrices are equally essential to promote their acceptance as healthy products. This study aims to explore 3D food printing technology to reinvent fermented and malted flours (traditionally processed edibles) as functional products with improved physical properties. Thus, the objectives were to investigate the color profile, browning index (BI), and structural properties of the 3D-printed and traditionally prepared snacks from whole-grain (WG) sourdough and malted flours.

## Methodology

### Preparation of raw, cowpea sourdough, and quinoa malt flours

Olenda variety cowpeas [(*Vigna unguiculata* (L.) Walp), Agricultural Research Council, Nelspruit, Mpumalanga, South Africa] and white quinoa [(*Chenopodium quinoa* Wild), Dis-Chem Retail pharmacy Pty (Ltd), Gauteng, South Africa)] were procured locally. The whole grains were cleaned off foreign materials, weighed, dry-milled (KJ-1250, Castelfranco Veneto, Italy), and sieved using a 500 µm mesh size (Analysette 3 Spartan, Fritsch, Germany) to obtain raw flours. A portion of the raw cowpea and quinoa flours served as control samples.

Cowpea sourdough and quinoa malt flours were selected in this study based on their complementary biochemical, nutritional, and techno-functional properties established in our previous study^12^. The reported procedure by Kewuyemi et al.^12^ was followed to prepare the cowpea sourdough and quinoa malt. Briefly, a portion of the WG cowpea flour was homogenously mixed with distilled water (1:1, w/v) and naturally fermented at 28 °C for 48 h (Labcon, Krugersdorp, South Africa). The cleaned quinoa grains were steeped in distilled water (1:3, w/v) for 24 h at 28 °C, drained, and germinated for 48 h at 28 °C. The recovered wet cowpea sourdough and sprouted quinoa grains were freeze-dried (Beijer Electronics HT40, Telstar LyoQuest, Terrassa, Spain), milled (KJ-1250, Castelfranco Veneto, Italy), and passed through a 500 µm mesh sieve (Analysette 3 Spartan, Fritsch, Germany) to obtain cowpea sourdough and quinoa malt flours.

### Processing of traditional and 3D-printed WG and composite biscuits

The dough formulations presented in Table 1 were used to prepare the traditional and 3D-printed biscuits. The higher level of cowpea sourdough to quinoa malt in the composite formulation is an approach to developing cowpea-based bakery products. The modified method described by Adebiyi et al.^13^ was followed to prepare the biscuits. Firstly, the dry ingredients [flours, Snowflake baking powder (Premier FMCG, Pty., Ltd., Johannesburg, South Africa), and Selati white sugar (RCL Foods Ltd., Durban, South Africa)] were mixed in a cleaned bowl. The dry mix was subsequently made into a cohesive dough by adding vegetable oil (Pick n Pay Retailers, Pty., Ltd., Kenilworth, South Africa), vanilla flavor (Libstar Operations Pty., Ltd., Plattekloof, South Africa), and water.

**Table 1:** Whole-grain and multigrain dough formulations used to prepare traditional and 3D-printed biscuits.

Ingredients	100:0	100:0	100:0	100:0	80:20	60:40
Raw cowpea flour (g)	70.70	0	0	0	0	0
Raw quinoa flour (g)	0	70.70	0	0	0	0
Cowpea sourdough flour (g)	0	0	70.70	0	56.56	42.42
Malted quinoa flour (g)	0	0	0	70.70	14.14	28.28
Water (mL)^a^	20.00 (20.00)	25.00 (28.80)	20.00 (32.00)	25.00 (32.00)	20.00 (30.20)	20.00 (28.00)
Sugar (g)	14.00	14.00	14.00	14.00	14.00	14.00
Sunflower oil (mL)	8.50	8.50	8.50	8.50	8.50	8.50
Vanilla flavour (g)	3.40	3.40	3.40	3.40	3.40	3.40
Baking powder (g)	0.40	0.40	0.40	0.40	0.40	0.40

^a^Distilled water levels (mL) in parenthesis were used to prepare the 3D-printed biscuits.

### Traditional biscuits preparation

The molded cohesive dough was manually kneaded on a flat surface into smooth dough sheets and cut using a cast into uniform sizes (38.00 mm: width, 52.50 mm: diameter, and 3.50 mm: height). The shaped doughs were packed in a stainless steel tray for baking.

### 3D model design and 3D printing process

**Figure 1:**
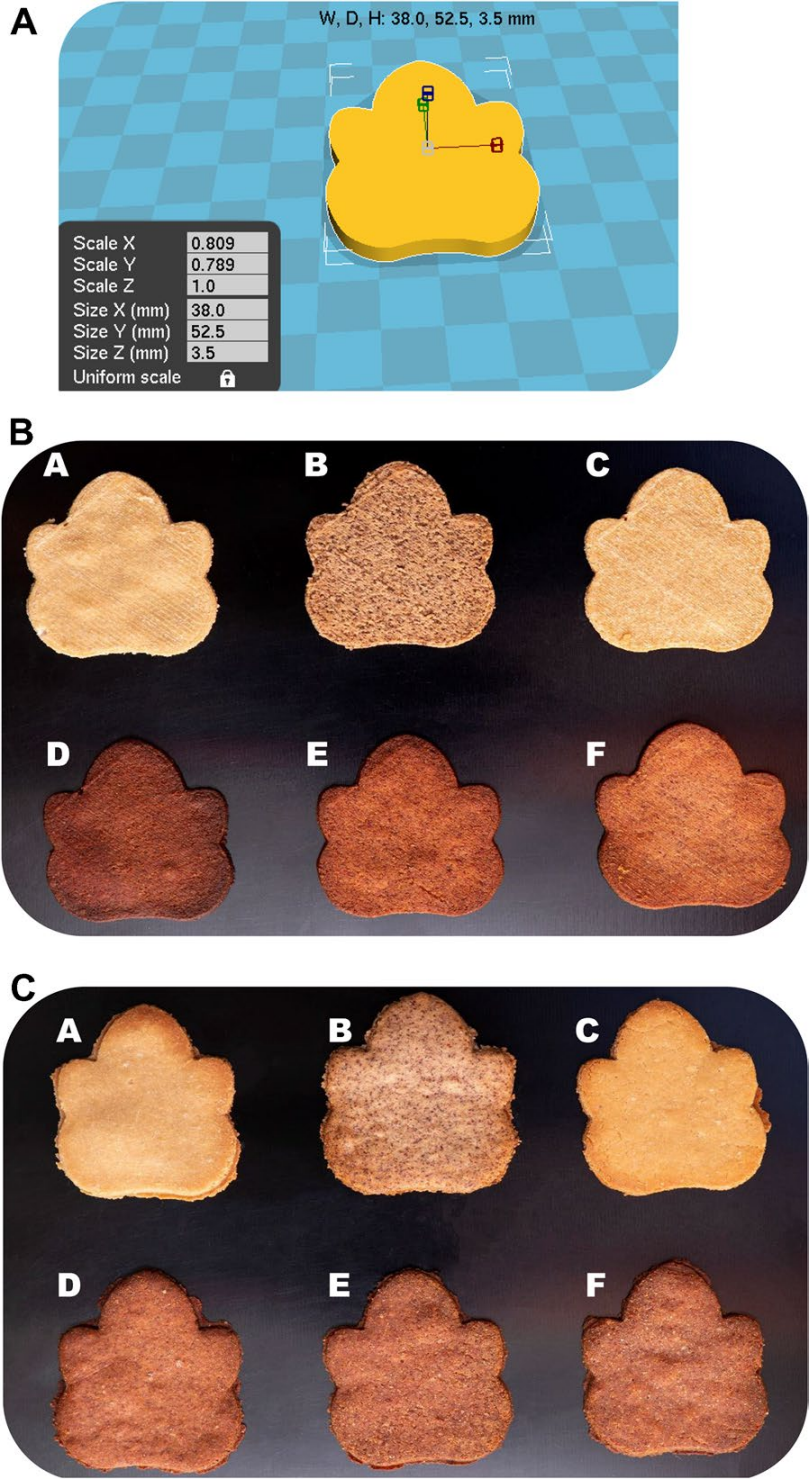
(**A**) The virtual three-dimensional (3D) model used for 3D-printing of prepared doughs. (**B**) Three-dimensionally (3D) printed and baked biscuits from raw, cowpea sourdough, and quinoa malt whole and multigrain flours. (**A**) Raw quinoa 3D-printed biscuit (RQ3D), (**B**) raw cowpea 3D-printed biscuit (RC3D), (**C**) quinoa malt 3D-printed biscuit (QM3D), (**D**) cowpea sourdough 3D-printed biscuit (CS3D), (**E**) 80% cowpea sourdough and 20% quinoa malt 3D-printed biscuit [CS-QM3D (80:20)], (**F**) 60% cowpea sourdough and 40% quinoa malt 3D-printed biscuit [CS-QM3D (60:40)]. (**C**) Traditional biscuits from raw, cowpea sourdough, and quinoa malt whole and multigrain flours. (**A**) Raw quinoa biscuit (RQB), (**B**) raw cowpea biscuit (RCB), (**C**) quinoa malt biscuit (QMB), (**D**) cowpea sourdough biscuit (CSB), (**E**) 80% cowpea sourdough and 20% quinoa malt biscuit [CS-QMB (80:20)] (**F**) 60% cowpea sourdough and 40% quinoa malt biscuit [CS-QMB (60:40)].

The dough formulations for 3D printing contained additional water (Table 1, water volume in parenthesis) to allow ease of extrusion. A multi-system comprising an Anycubic Delta 3D printer (Anycubic D, Shenzhen Anycubic Technology Co., Ltd., Shenzhen, China), an oil-free air compressor (EWS06-2, MAC-AFRIC, Johannesburg, South Africa), and dispensing controller (983A, USA Technology, and Materials, Shanghai, China) was modified for the 3D food printing. Prior to the printing process, a computer-aided design (Fig. 1A), saved as a stereolithography *(STL)* extension file, was designed on *Meshmixer* (version 11.0.544, Autodesk Incorporation, San Rafael, USA) and sliced using *Cura* (ver. 15.04.6, Ultimaker B.V., Utrecht, The Netherlands) to obtain a geometric code (*g-code*) extension file. The generated *g-code* file was copied to the modified printer using a secure digital card for the food printing. The prepared cohesive dough at room temperature was aseptically half-filled in the printer syringe barrels without air bubbles and extruded at ambient temperature using the supplied air pressure ( ≈6 bar). The preliminarily established best printing conditions: ink flow (100%), nozzle height (0.59 mm), nozzle diameter (0.50 mm), and printing speed (10 mm/s) were used. The 3D-printed doughs were instantly transferred into a freezer preset at −21 °C for 2 h [KBF 631, KIC SA (Pty) Ltd., Johannesburg, South Africa] to maintain structural integrity.

### Baking of traditionally prepared and 3D-printed doughs

The baking oven (Macadams International, Pty., Ltd., Johannesburg, South Africa) was heated for 1 h at the desired temperature of 180 °C and further stabilized for another 2 h prior to baking the shaped doughs. The traditionally prepared and 3D-printed doughs were flatly placed on two baking trays. Each group (traditional or 3D-printed doughs) were baked in the preheated oven at 180 °C for 15 ± 2 min. During baking, the oven temperature was monitored via the fitted oven thermometer gauge to ensure 180 °C was maintained throughout the cooking process. The baked biscuits were cooled for 2 h and subsequently weighed [traditional biscuits (7 ± 0.2 g) and 3D-printed biscuits (6 ± 0.2 g)]. Then, the snacks were examined for physical properties. The moisture contents (934.01^14^) of the freeze-dried (Beijer Electronics HT40, Telstar LyoQuest, Terrassa, Spain) biscuits at − 55 °C for 24 h (to prevent surrounding moisture interference)] were also determined.

### Color profile of the traditional and 3D-printed biscuits

The color parameters of the prepared biscuits (top and bottom views) were evaluated using chroma meter-410 connected with a Data Procesor-400 (Ver. 1.20) (Konica Minolta, Inc., Tokyo, Japan). Appropriate calibration was done using the supplied white tiles (Y – 85.0, x – 0.3165, and y – 0.3231) and the color profiles: L*—lightness, a*—redness, b*—yellowness, ∆E* (CIELAB), and ∆E (Hunter lab)—total color differences were recorded in triplicates. The snacks browning indexes were computed using Eqs. (1) and (2)^15^.

1 $$\text{Browning index = }\frac{\left[{100}\left(\text{y }- 0.31\right)\right]}{0.17}$$

2 $$\text{y = }\frac{\text{(}{\text{a}}^{*}\text{ + 1.75}{\text{L}}^{*}\text{)}}{\left(\text{5.645}{\text{L}}^{*}\text{ + }{\text{a}}^{*}{ - 3.012}{\text{b}}^{*}\right)}$$

### Image analyses of the traditional and 3D-printed biscuits

The C-Cell food imaging system (Calibre, Warrington, United Kingdom) interfaced with *C-Cell* software was used to provide the biscuits’ structural analysis. The manufacturer guide was followed to estimate the structural parameters. Parameters such as area of cells (%), average cell elongation, cell diameter (mm), cell volume, wall thickness (mm), and total concavity (%) were recorded in triplicates. The average cell area (mm^2^) was estimated by dividing the total snack area by the number of cells. All data were obtained in triplicates after standardizing the equipment with the attached calibration white card for accuracy.

### Texture (hardness) analysis of the traditional and 3D-printed biscuits

The hardness of the biscuits was measured using the procedure by Derossi et al.^5^ with minor modifications. A 70 mm aluminum break probe (671170) and three-point bend rig (675040) fitted to the base of a food texture analyzer (TVT 6700, Perten instruments, Hägersten, Sweden) was employed. The machine was interfaced via *TexCalc* software (ver. 4.0.4.67), and the operating conditions include: compression (70%), initial speed (2.0 mm/s), retract speed (10.0 mm/s), test speed (3.0 mm/s), and trigger force (5 g). At least six runs were experimented on per sample. The hardness of the traditional and 3D-printed biscuits was estimated as the force required to trigger a given deformation, and it was expressed as the peak force in Newton.

### Statistical analysis

The data generated in triplicates were subjected to analysis of variance (one-way ANOVA) using a statistical software package (IBM SPSS, ver. 27.0, New York, USA). A Duncan multiple range test was selected to determine the significant differences (p ≤ 0.05) between the snack samples. The obtained results are shown as mean values and standard deviations.

### Statement on experimental research and field studies on plants

The authors confirmed that the use of a plant-based pulse legume and pseudocereal in our study complied with the relevant institutional, national, and international guidelines and legislation, in particular, the IUCN Policy Statement on Research Involving Species at Risk of Extinction.

## Results and discussion

### Comparisons between the virtual three-dimensional (3D) model, 3D-printed, and traditional biscuits

The computer-aided design and 3D-printed biscuits’ images are depicted in Fig. 1A,B, respectively. The 3D-printed biscuits were typically observed to replicate the virtual model, with no compressed deformation and fewer defect points. Although the traditional and 3D-printed biscuits were prepared using the same ingredients, visual comparisons (Fig. 1B,C) revealed that the 3D-printed biscuits showed considerable distinction in terms of consistent structural appearance and color intensities. The desirable characteristics of the 3D-printed biscuits may enhance the acceptability of snack foods and reinvent locally prepared meals as trendy, sustainable, and functional foods.

### Color profile and browning index of the traditional and 3D-printed biscuits

Tables 2 and 3 summarize the color profile and browning index of the raw, cowpea sourdough, quinoa malt whole- and multigrain biscuits. The traditional and 3D-printed snacks’ surfaces (a) top view and (b) bottom view were examined for the color test. The result revealed that the top and bottom views of the snacks had similar trends of color intensities. This observation may suggest that the baking doughs were evenly cooked. Significant differences (p ≤ 0.05) in the color parameters of the snacks with different flour formulations were also noted. The biscuits’ color differences were in harmony with the distinct appearances of their based-flours reported in an earlier investigation^12^. It is worthy to note that the cowpea sourdough and malted quinoa flours-based bioprocesses (fermentation and malting) involve the hydrolysis of major polymer components (protein and starch) into probably elevated concentrations of monomers such as amino acids and reducing sugars^13,16^. These derived bioproducts might have contributed to a more profound caramelization and Maillard reactions during the baking of the snacks, especially in malted quinoa snacks^13,17^. Furthermore, the visual differences of the same snack formulations (especially between cowpea sourdough and its composite snacks) differed by preparation mode (Fig. 1B,C) can be ascribed to the smooth-like surface induced by manual dough kneading and deposited strands of dough stacked in layers through 3D-printing^6^.

**Table 2:** Color profile of the raw, cowpea sourdough, quinoa malt whole and multigrain biscuits.

Sample	Top view						Bottom view					
	L*	a*	b*	∆E*	∆E	BI	L*	a*	b*	∆E*	∆E	BI
RCB	52.98^d^ [0]	4.22^a^ [0]	7.58^c^ [0.01]	0.01^a^ [0]	0.01^a^ [0.01]	20.95^a^ [0.01]	52.41^d^ [0.01]	4.51^a^ [0.02]	7.62^c^ [0.02]	0.02^a^ [0.02]	0.01^a^ [0.01]	21.70^a^ [0.03]
RQB	62.16^e^ [0.01]	5.85^b^ [0.02]	18.69^e^ [0.01]	0.01^a^ [0.02]	0.05^b^ [0.01]	42.08^e^ [0.03]	61.26^e^ [0.01]	6.53^b^ [0.01]	19.07^e^ [0.01]	0.02^a^ [0.02]	0.02^a^ [0]	44.51^e^ [0]
CSB	48.67^b^ [0.01]	8.12^d^ [0.01]	6.86^a^ [0.01]	5.86^c^ [0.02]	6.31^d^ [0.02]	26.93^b^ [0.02]	48.27^a^ [0.01]	8.22^d^ [0.02]	6.29^a^ [0.02]	5.70^c^ [0.02]	4.88^d^ [0.01]	25.96^b^ [0.01]
QMB	63.08^f^ [0.01]	6.29^c^ [0.02]	20.47^f^ [0.01]	2.05^b^ [0.01]	1.59^c^ [0.01]	45.88^f^ [0.01]	62.46^f^ [0.02]	6.98^c^ [0.03]	20.60^f^ [0.01]	1.99^b^ [0.01]	1.04^b^ [0]	47.56^f^ [0.03]
CS-QMB (80:20)	48.98^c^ [0.01]	8.52^f^ [0.02]	7.93^d^ [0.01]	5.88^d^ [0.01]	6.36^e^ [0.02]	29.93 [0.02]	48.54^b^ [0.01]	8.68^e^ [0.02]	7.39^b^ [0.01]	5.68^c^ [0.02]	4.80^c^ [0.02]	29.13^c^ [0.02]
CS-QMB (60:40)	48.37^a^ [0.01]	8.36^e^ [0.01]	7.44^b^ [0.01]	6.20^e^ [0]	6.79^f^ [0.01]	28.88^c^ [0.02]	48.69^c^ [0.01]	8.83^f^ [0.03]	7.79^d^ [0.01]	5.69^c^ [0.02]	5.21^e^ [0.03]	30.24^d^ [0.02]

Various superscripts presented per column indicate significant differences (*p* ≤ 0.05). Values in square brackets are standard deviations of the respective means.

L* lightness, a* redness, b* yellowness, ∆E* total color difference for CIELAB coordinates, *∆E* total color difference for Hunter LAB coordinates, *BI* browning index, *RCB* raw cowpea biscuit, *RQB* raw quinoa biscuit,* CSB* cowpea sourdough biscuit, *QMB* quinoa malt biscuit,* CS-QMB (80:20)* 80% cowpea sourdough and 20% quinoa malt biscuit, *CS-QMB (60:40)* 60% cowpea sourdough and 40% quinoa malt biscuit.

**Table 3:** Color profile of the raw, cowpea sourdough, quinoa malt whole and multigrain 3D-printed biscuits.

Sample	Top view						Bottom view					
	L*	a*	b*	∆E*	∆E	BI	L*	a*	b*	∆E*	∆E	BI
RC3D	55.22^d^ [0.01]	4.54^a^ [0.01]	8.27^d^ [0.01]	0.01^a^ [0]	0.01^a^ [0.01]	21.93^a^ [0.03]	56.26^d^ [0.01]	5.39^b^ [0.02]	9.89^d^ [0.01]	0.02^a^ [0.02]	0.02^a^ [0.01]	26.00^c^ [0.03]
RQ3D	63.11^f^ [0.01]	5.20^b^ [0.01]	16.99^e^ [0.02]	0.02^a^ [0.01]	0.04^b^ [0.01]	36.89^e^ [0.02]	63.28^f^ [0.01]	5.07^a^ [0.01]	17.05^e^ [0.01]	0.04^b^ [0.01]	0.02^a^ [0.02]	36.74^e^ [0.01]
CS3D	47.35^a^ [0.02]	7.35^e^ [0.01]	5.31^a^ [0.01]	8.87^e^ [0.02]	8.72^f^ [0.01]	22.82^b^ [0.02]	48.24^a^ [0]	7.60^d^ [0.01]	5.74^a^ [0.01]	9.31^f^ [0.01]	9.95^e^ [0.01]	23.76^a^ [0.03]
QM3D	61.94^e^ [0.01]	5.39^c^ [0.01]	17.41^f^ [0.01]	1.26^b^ [0.01]	3.02^c^ [0.01]	38.84^f^ [0.02]	62.01^e^ [0.02]	5.55^c^ [0.03]	17.52^f^ [0.02]	1.44^c^ [0.01]	1.47^b^ [0.01]	39.22^f^ [0.02]
CS-QM3D (80:20)	48.45^b^ [0.01]	7.33^d^ [0.01]	6.31^b^ [0.01]	7.58^d^ [0.01]	7.01^e^ [0.01]	24.60^c^ [0.03]	49.47^b^ [0.01]	7.58^d^ [0.01]	6.58^b^ [0.02]	7.88^e^ [0.01]	8.63D [0.01]	25.05^b^ [0.03]
CS-QM3D (60:40)	49.40^c^ [0.01]	7.63^f^ [0.02]	8.03^c^ [0.01]	6.59^c^ [0.02]	6.10^d^ [0.01]	28.62^d^ [0.02]	50.75^c^ [0.01]	7.65^e^ [0.02]	7.99^c^ [0.01]	6.26^d^ [0.01]	7.09^c^ [0.01]	27.75^d^ [0.01]

Various superscripts presented per column indicate significant differences (*p* ≤ 0.05). Values in square brackets are standard deviations of the respective means.

L* lightness, a* redness, b* yellowness, ∆E* total color difference for CIELAB coordinates, *∆E* total color difference for Hunter LAB coordinates, *BI* browning index, *3D* three-dimensional, *RC3D* raw cowpea 3D-printed biscuit, *RQ3D* raw quinoa 3D-printed biscuit, *CS3D* cowpea sourdough 3D-printed biscuit, *QM3D* quinoa malt 3D-printed biscuit, *CS-QM3D (80:20)* 80% cowpea sourdough and 20% quinoa malt 3D-printed biscuit, *CS-QM3D (60:40)* 60% cowpea sourdough and 40% quinoa malt 3D-printed biscuit.

Among the traditional snacks, the top and bottom views of the quinoa malt snack had the highest lightness (63.08 and 62.46), yellowness (20.47 and 20.60), and browning index (45.88 and 47.56), respectively. Correspondingly, the 3D-printed malted quinoa snack displayed similar trends of lightness (61.94 and 62.01), yellowness (17.41 and 17.52), and browning index (38.84 and 39.22). By contrast, the traditional and 3D-printed snacks containing cowpea sourdough indicated the highest redness (8.12/8.22 and 7.35/7.60, respectively) and color space difference (4.88–6.31 and 8.72–9.95, respectively) values compared with the raw and quinoa malt snacks. The highest lightness and yellowness recorded for quinoa malt snacks reflect their respective intensities in the raw quinoa snacks. The observed redness increase (4.22–8.22 and 4.54–7.60) in the cowpea sourdough snacks, which influenced higher color difference values, could be ascribed to the higher protein content of the snacks that enhanced the Maillard reaction^18^. Interestingly, the multigrain snacks comprising cowpea sourdough and quinoa malt might have shown a better ratio of reducing sugars to amino compounds and thus facilitated improved a* through caramelization and Maillard reactions and inconsistent increases and decreases in the lightness and yellowness of the snacks^17^.

The increased redness level noted for the traditional and 3D-printed cowpea sourdough snacks would have been expected to reflect in the browning index (26.93/25.96 and 22.82/23.76, respectively) of the snacks. However, the overall low browning index of cowpea sourdough snacks may plausibly be due to the high acidity level of cowpea sourdough flour^12^, resulting in a deleterious effect on its nucleophilicity. The nucleophilic attack of the primary amino group of a nitrogen-based compound on the carbonyl group is required to trigger the Maillard reaction^19^. Amaya-Farfan and Rodriguez-Amaya^19^ highlighted that protonated amino groups (formed due to the pH drop of amino group isoelectric point below neutral pH 6–7) are not able to be involved in nucleophilic reactions. Therefore, a lower degree of the Maillard reaction (browning index) in cowpea sourdough snacks.

### Image analyses of the traditional and 3D-printed biscuits

The image analyses of the traditional and 3D-printed biscuits are reported in Tables 4, 5, 6 and 7. The data relates the link between processing mode variations and the corresponding effects on the resultant traditional and 3D-printed biscuits’ structural features. The crumb structure (interior part of the biscuits) essentially comprises the fluid phase (air cells or pores) and solid phase (cell wall)^20,21^. The air cells span a wide range of microns within the crumb^20^. The estimated biscuits’ cellular and physical characteristics are broadly grouped into cell size (area of cells, average cell area, cell diameter, cell volume, and wall thickness), cell elongation (average cell elongation), and the biscuit shape (total concavity). The cell size and elongation describe the cell information in terms of cell area, average cell elongation, cell diameter, cell volume, and wall thickness within the crumb. The biscuits shape is represented as the total concavity extent of the biscuits.

**Table 4:** Image analyses of the raw, cowpea sourdough, quinoa malt whole and multigrain biscuits.

Sample	Area of cells (%)	Average cell area (mm^2^)	Average cell elongation	Cell diameter (mm)	Cell volume	Wall thickness (mm)	Total concavity (%)
RCB	45.33^ab^ [0.15]	0.74^b^ [0.01]	1.46^a^ [0]	1.17^a^ [0.02]	3.24^c^ [0.09]	0.34^b^ [0]	5.21^b^ [0.01]
RQB	49.30^d^ [0.10]	0.73^b^ [0.05]	1.48^b^ [0.01]	1.27^b^ [0.08]	2.26^ab^ [0.31]	0.31^a^ [0.01]	3.81a [0.61]
CSB	46.43^bc^ [0.21]	0.61^a^ [0.02]	1.49^b^ [0.01]	1.14^a^ [0.07]	2.32^b^ [0.13]	0.31^a^ [0]	5.38^b^ [0.38]
QMB	47.17^c^ [0.84]	0.61^a^ [0.01]	1.51^c^ [0.01]	1.16^a^ [0.03]	1.92^a^ [0.12]	0.31^a^ [0]	4.19^a^ [0.48]
CS-QMB (80:20)	46.13^bc^ [0.12]	0.58^a^ [0]	1.49^b^ [0.01]	1.15^a^ [0.01]	2.35^b^ [0.11]	0.31^a^ [0]	5.30^b^ [0.04]
CS-QMB (60:40)	44.93^a^ [1.29]	0.57^a^ [0.03]	1.49^b^ [0.01]	1.12^a^ [0.08]	2.22^ab^ [0.25]	0.31^a^ [0]	4.23^a^ [0.49]

Various superscripts presented per column indicate significant differences (*p* ≤ 0.05). Values in square brackets are the standard deviations of the respective means.

*RCB* raw cowpea biscuit, *RQB* raw quinoa biscuit, *CSB* cowpea sourdough biscuit, *QMB* quinoa malt biscuit, *CS-QMB (80:20)* 80% cowpea sourdough and 20% quinoa malt biscuit, *CS-QMB (60:40)* 60% cowpea sourdough and 40% quinoa malt biscuit.

**Table 5:** Image analyses of the raw, cowpea sourdough, quinoa malt whole and multigrain 3D-printed biscuits.

Sample	Area of cells (%)	Average cell area (mm^2^)	Average cell elongation	Cell diameter (mm)	Cell volume	Wall thickness (mm)	Total concavity (%)
RC3D	46.20^c^ [0.35]	0.88^f^ [0.01]	1.53^ab^ [0.01]	1.19^d^ [0.01]	4.02^e^ [0.01]	0.36^a^ [0]	5.23^c^ [0]
RQ3D	50.60^d^ [0.30]	0.79^e^ [0.01]	1.54^c^ [0]	1.22^d^ [0.03]	2.77^d^ [0.07]	0.32^a^ [0]	5.19^c^ [0.05]
CS3D	43.60^a^ [0.10]	0.57^a^ [0.01]	1.53^ab^ [0.01]	0.93^a^ [0.03]	2.21^a^ [0.01]	0.31^a^ [0]	5.03^b^ [0.04]
QM3D	50.57^d^ [0.71]	0.76^d^ [0.01]	1.52^b^ [0.01]	1.32^e^ [0.02]	2.37^b^ [0.05]	0.32^a^ [0]	4.91^a^ [0.04]
CS-QM3D (80:20)	45.17^b^ [0.40]	0.64^c^ [0]	1.50^a^ [0.01]	1.05^c^ [0.05]	2.62^c^ [0.17]	0.32^a^ [0]	5.01^b^ [0.04]
CS-QM3D (60:40)	44.50^b^ [0.10]	0.62^b^ [0]	1.53^ab^ [0.01]	0.99^b^ [0.02]	2.39^b^ [0.02]	0.32^a^ [0]	4.92^a^ [0.05]

Various superscripts presented per column indicate significant differences (*p* ≤ 0.05). Values in square brackets are the standard deviations of the respective means.

*3D* three-dimensional, *RC3D* raw cowpea 3D-printed biscuit, *RQ3D* raw quinoa 3D-printed biscuit, *CS3D* cowpea sourdough 3D-printed biscuit, *QM3D* quinoa malt 3D-printed biscuit, *CS-QM3D (80:20)* 80% cowpea sourdough and 20% quinoa malt 3D-printed biscuit, *CS-QM3D (60:40)* 60% cowpea sourdough and 40% quinoa malt 3D-printed biscuit.

**Table 6:**
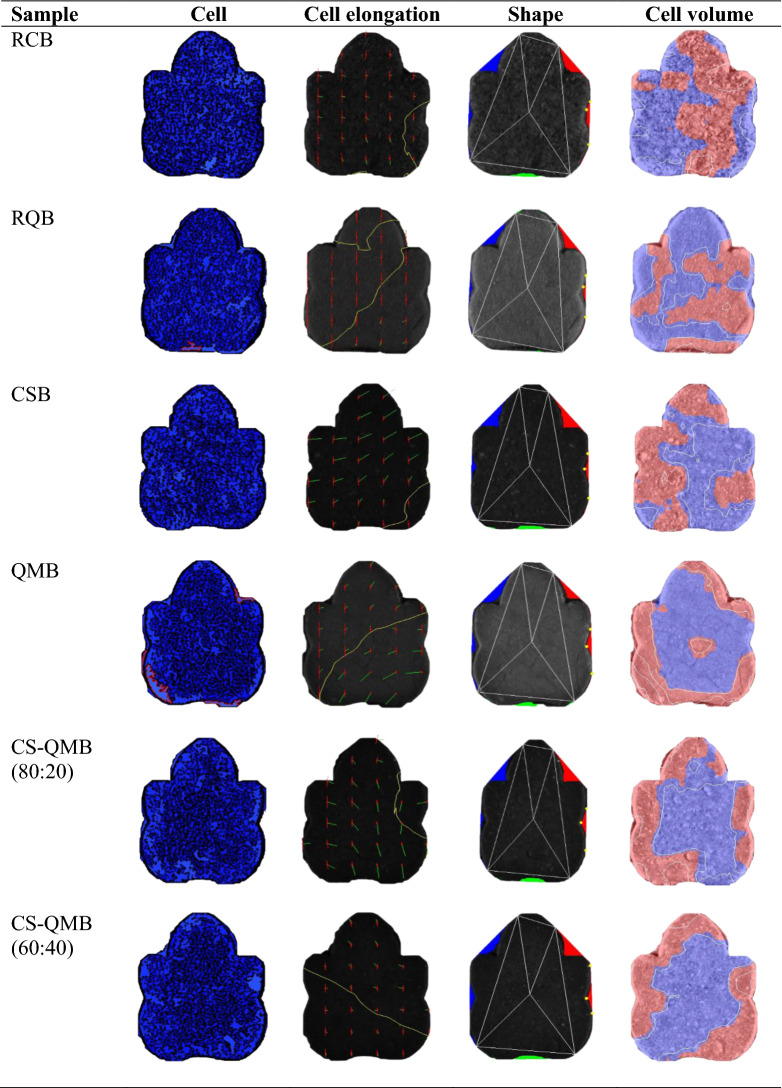
Structural images of the traditional biscuits generated via a digital imaging system (C-Cell).

*RCB* raw cowpea biscuit, *RQB* raw quinoa biscuit, *CSB* cowpea sourdough biscuit, *QMB* quinoa malt biscuit, *CS-QMB (80:20)* 80% cowpea sourdough and 20% quinoa malt biscuit, *CS-QMB (60:40)* 60% cowpea sourdough and 40% quinoa malt biscuit.

**Table 7:**
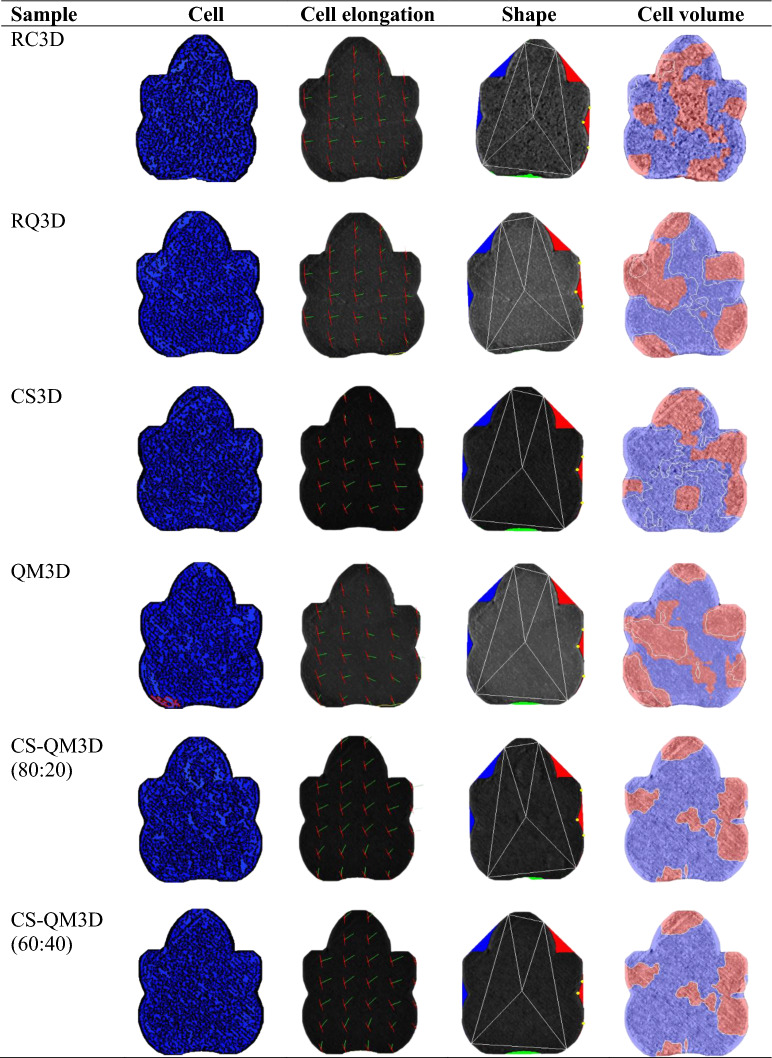
Structural images of the 3D-printed biscuits generated via a digital imaging system (C-Cell).

*3D* three-dimensional, *RC3D* raw cowpea 3D-printed biscuit, *RQ3D* raw quinoa 3D-printed biscuit, *CS3D* cowpea sourdough 3D-printed biscuit, *QM3D* quinoa malt 3D-printed biscuit, *CS-QM3D (80:20)* 80% cowpea sourdough and 20% quinoa malt 3D-printed biscuit, *CS-QM3D (60:40)* 60% cowpea sourdough and 40% quinoa malt 3D-printed biscuit.

The traditionally prepared cowpea sourdough, quinoa malt, and multigrain snacks showed nonsignificant differences (p ≤ 0.05) for the cell size data except for slight differences (p ≤ 0.05) in the area of cells and cell volume. The cell size [area of cells (44.93–47.17%), average cell area (0.57–0.61 mm^2^), cell diameter (1.12–1.16 mm), cell volume (1.92–2.35), and wall thickness (0.31 mm)] of these snacks was observed to be generally low compared to the raw snacks’ cell size [area of cells (45.33–49.30%), average cell area (0.73–0.74 mm^2^), cell diameter (1.17–1.27 mm), cell volume (2.26–3.24) and wall thickness (0.31–0.34 mm), respectively]. Similar trends of the traditional snacks’ cell size data were also noted for the 3D-printed snacks. The observed cell size decrease in the cowpea sourdough, quinoa malt, and composite snacks may be due to their dough acidification by the modified-based flours^12^. The dough acidification has been suggested to have a linear impact on cell formation and size by modifying the interaction between moisture and biomolecules in the snack formulations^22,23^. Additionally, the increase in the crude fat levels of cowpea sourdough and quinoa malt flours^12^ might have caused a simultaneous reduction in the air cells and enhanced cell coalescence of the biscuits^21^.

Further comparisons of the cell size values between the snacks revealed that the 3D-printed biscuits generally had higher averaged cell area (0.57–0.88 mm^2^), cell volume (2.21–4.02), and wall thickness (0.31–0.36 mm) compared to the traditional biscuits (0.57–0.74 mm^2^, 1.92–3.24, 0.31–0.34 mm, respectively). The contrasting cellular architectures of the biscuits are in accordance with differences in microstructural data reported for baked (biscuits and wafer) and conventionally extruded products^24,25^. The cellular changes were linked to the distinct impact of the processing modes. Specifically, added moisture in 3D-printed biscuits could have resulted in a denser baked product with thicker cell walls^25^.

Regarding the average cell elongation of the snacks, the 3D-printed snacks showed higher values (1.50–1.54) than the traditional snacks (1.46–1.51). The extended cell elongation of the 3D-printed snacks may be related to the modification of biomolecules due to the induced in-barrel dough shearing effect and stepwise deposition of stacked dough strands during printing^26^. Also, the slight increases in the water level (Table 1) of the 3D-printed snacks to obtain a less viscous dough for printability might have contributed to the elongated cells compared to those of the traditionally prepared snacks. The physical significance of the total concavities of the biscuits illustrates the inward deviation extent in the biscuits’ shape. The 3D-printed snacks showed a slight deviation in the total concavities (4.91–5.23%), whereas the traditional snacks showed a higher deviation in the total concavity values (3.81–5.38%). The lower variation in the 3D-printed snacks’ total concavities suggests the consistency in replicating layers of the printed snacks.

### Hardness of the traditional and 3D-printed biscuits

**Figure 2:**
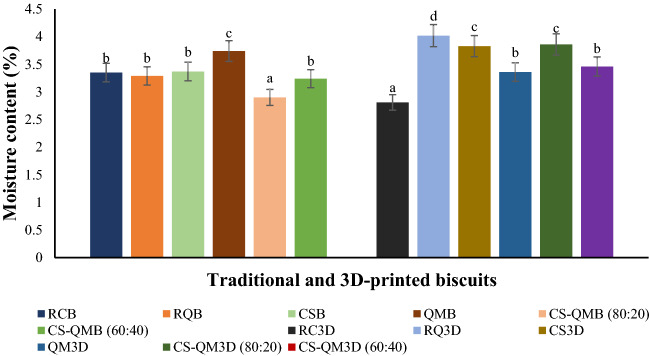
Moisture content of the raw, cowpea sourdough, and quinoa malt whole and multigrain biscuits. Averages of the moisture content depicted as bars with superscripts indicate significant differences (*p* ≤ 0.05). *RCB* raw cowpea biscuit, *RQB* raw quinoa biscuit, *CSB* cowpea sourdough biscuit, *QMB* quinoa malt biscuit, *CS-QMB* (*80:20*) 80% cowpea sourdough and 20% quinoa malt biscuit, *CS-QMB* (*60:40*) 60% cowpea sourdough and 40% quinoa malt biscuit. *3D* three-dimensional, *RC3D* raw cowpea 3D-printed biscuit, *RQ3D* raw quinoa 3D-printed biscuit, *CS3D* cowpea sourdough 3D-printed biscuit, *QM3D* quinoa malt 3D-printed biscuit, *CS-QM3D* (*80:20*) 80% cowpea sourdough and 20% quinoa malt 3D-printed biscuit, *CS-QM3D (60:40)* 60% cowpea sourdough and 40% quinoa malt 3D-printed biscuit.

**Figure 3:**
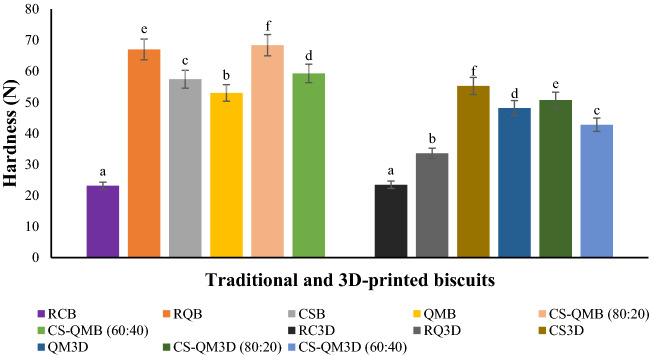
Texture (hardness) properties of the raw, cowpea sourdough, and quinoa malt whole and multigrain biscuits. Averages of the hardness depicted as bars with superscripts indicate significant differences (*p* ≤ 0.05). *RCB* raw cowpea biscuit, *RQB* raw quinoa biscuit, *CSB* cowpea sourdough biscuit, *QMB* quinoa malt biscuit, *CS-QMB (80:20)* 80% cowpea sourdough and 20% quinoa malt biscuit, *CS-QMB* (*60:40*) 60% cowpea sourdough and 40% quinoa malt biscuit, *3D* three-dimensional, *RC3D* raw cowpea 3D-printed biscuit, *RQ3D* raw quinoa 3D-printed biscuit, *CS3D* cowpea sourdough 3D-printed biscuit, *QM3D* quinoa malt 3D-printed biscuit, *CS-QM3D (80:20)* 80% cowpea sourdough and 20% quinoa malt 3D-printed biscuit, *CS-QM3D (60:40)* 60% cowpea sourdough and 40% quinoa malt 3D-printed biscuit.

It should be noted that the need for added water in the 3D-printed doughs and printing dough strands in layers led to slightly higher moisture contents (Fig. 2) and low weight (6 ± 0.2 g) of the derived printed biscuits. There were significant differences (p ≤ 0.05) in the hardness values of the produced biscuits (Fig. 3). The traditional snacks were observed to exhibit higher hardness (23.14–68.38 N) than the 3D-printed snacks (23.47–55.26 N). Although Derossi et al.^6^ demonstrated that the 3D food printing process intrinsically led to the creation of bigger pores but less in number and like-round in shape, which caused high hardness of the obtained printed snacks compared to the traditional snacks (both snacks were prepared from dough with similar weight). In this study, the slight increase in the 3D-printed biscuits’ moisture level (Fig. 2) and weight decrease might have contributed to low hardness values (i.e., low penetration force). Interestingly, the 3D-printed biscuits displayed a larger average air cell area and cell volume (Table 5) that could have reduced the biscuits’ hardness^27^. Nevertheless, the slightly low hardness values of the printed snacks may be desirable for ease of mastication.

## Conclusion

Traditional and 3D food printing techniques were employed in this study to investigate structural variations in the resultant WG and multigrain biscuits. The traditional and 3D-printed biscuits indicated considerable differences (p ≤ 0.05) in the color profile, browning index, image parameters, and texture (hardness). Besides the biscuits’ unique flour formulation impact, the 3D-printed biscuits exhibited distinct color intensities and improved consistency of structural design. The traditionally prepared cowpea sourdough, quinoa malt, and composite biscuits had higher hardness values than the 3D-printed counterparts. A combination of automated mechanical steps involved in restructuring the printed dough in layers and baking might have resulted in differences in the physical and cellular structure characteristics. The 3D-printed composite biscuits are suggested to be more desirable based on their multi flour formulation, low redness, browning index, and hardness. The printed composite biscuits also showed less variation in the total concavity and improved cell volume, average cell area, and average cell elongation. To enhance the acceptability of the edible composite 3D-printed biscuits, further studies are recommended to prepare the traditional and 3D-printed biscuits using the same water content and examine the biscuits’ structural, dimensional and cooking characteristics, sensory profile, and shelf stability.

## Data Availability

The data obtained in this study are available from the corresponding author on reasonable request.

## References

[1] Escalante-Aburto A, Trujillo-de Santiago G, Álvarez M, Chuck-Hernández C (2021). Advances and prospective applications of 3D food printing for health improvement and personalized nutrition. Compr. Rev. Food Sci. Food Saf..

[2] Kewuyemi Y, Kesa H, Adebo O (2021). Trends in functional food development with three-dimensional (3D) food printing technology: Prospects for value-added traditionally processed food products. Crit. Rev. Food Sci. Nutr..

[3] Oral M, Derossi A, Caporizzi R, Severini C (2021). Analyzing the most promising innovations in food printing. Programmable food texture and 4D foods. Future Foods.

[4] Derossi A, Caporizzi R, Paolillo M, Oral M, Severini C (2021). Drawing the scientific landscape of 3D food printing. Maps and interpretation of the global information in the first 13 years of detailed experiments, from 2007 to 2020. Innov. Food Sci. Emerg. Technol..

[5] Derossi A, Caporizzi R, Paolillo M, Severini C (2021). Programmable texture properties of cereal-based snack mediated by 3D printing technology. J. Food Eng..

[6] Derossi A, Caporizzi R, Oral M, Severini C (2020). Analyzing the effects of 3D printing process per se on the microstructure and mechanical properties of cereal food products. Innov. Food Sci. Emerg. Technol..

[7] Zhang L, Noort M, VanBommel K (2021). Towards the creation of personalized bakery products using 3D food printing. Adv. Food Nutr. Res..

[8] Cotabarren, I. & Palla, C. Development of functional foods by using 3D printing technologies: application to oxidative stress and inflammation-related affections. in *Current Advances for Development of Functional Foods Modulating Inflammation and Oxidative Stress* (ed. Hernández-Ledesma, B. & Martínez-Villaluenga, C.). 33–55. (Elsevier, 2022).

[9] Portanguen, S., Tournayre, P., Sicard, J., Astruc, T. & Mirade, P. 3D food printing: Genesis, trends and prospects. in *Global Trends, Opportunities, and Sustainability Challenges* (ed. Bhat, R.). 627–644. (Elsevier, 2022).

[10] Hussain S, Malakar S, Arora V (2022). Extrusion-based 3D food printing: Technological approaches, material characteristics, printing stability, and post-processing. Food Eng. Rev..

[11] He C, Zhang M, Fang Z (2020). 3D printing of food: Pretreatment and post-treatment of materials. Crit. Rev. Food Sci. Nutr..

[12] Kewuyemi YO, Kesa H, Adebo OA (2022). Biochemical properties, nutritional quality, colour profile and techno-functional properties of whole grain sourdough and malted cowpea and quinoa flours. Int. J. Food Sci. Technol..

[13] Adebiyi JA, Obadina AO, Adebo OA, Kayitesi E (2017). Comparison of nutritional quality and sensory acceptability of biscuits obtained from native, fermented, and malted pearl millet (*Pennisetum glaucum*) flour. Food Chem..

[14] AOAC. *Official Methods of Analysis. Proximate Analysis*. 17th edn. (Association of Analytical Communities, NITR, NT, 2006).

[15] Emerald FM (2020). Modelling approaches for predicting moisture transfer during baking of *chhana podo* (milk cake) incorporated with *tikhur* (*Curcuma angustifolia*) starch. J. Food Meas. Charact..

[16] Yang B, Yin Y, Liu C, Zhao Z, Guo M (2021). Effect of germination time on the compositional, functional and antioxidant properties of whole wheat malt and its end-use evaluation in cookie-making. Food Chem..

[17] Yang B, Guo M, Zhao Z (2020). Incorporation of wheat malt into a cookie recipe and its effect on the physicochemical properties of the corresponding dough and cookies. Lebensm. Wiss. Technol..

[18] Chinma C (2020). Evaluation of fermented African yam bean flour composition and influence of substitution levels on properties of wheat bread. J. Food Sci..

[19] Amaya-Farfan, J. & Rodriguez-Amaya, D. B. The Maillard reactions. in *Chemical Changes During Processing and Storage of Foods: Implications for Food Quality and Human Health* (ed. Rodriguez-Amaya, D. B. & Amaya-Farfan, J.). 215–263. (Elsevier, 2021).

[20] Farrera-Rebollo RR (2012). Evaluation of image analysis tools for characterization of sweet bread crumb structure. Food Bioproc. Tech..

[21] Dewaest M (2018). Effect of crumb cellular structure characterized by image analysis on cake softness. J. Texture Stud..

[22] Habibi Najafi M, Pourfarzad A, Zahedi H, Ahmadian-Kouchaksaraie Z, Haddad Khodaparast M (2016). Development of sourdough fermented date seed for improving the quality and shelf life of flat bread: Study with univariate and multivariate analyses. J. Food Sci. Technol..

[23] Longoria S, Contreras J, Belmares R, Cruz M, Flores M (2020). Effect of short fermentation times with *Lactobacillus paracasei* in rheological, physical and chemical composition parameters in cassava dough and biscuits. Appl. Sci..

[24] Chevallier S, Réguerre A, Le Bail A, Della Valle G (2014). Determining the cellular structure of two cereal food foams by X-ray micro-tomography. Food Biophys..

[25] Butt S (2018). Quantifying the differences in structure and mechanical response of confectionery products resulting from the baking and extrusion processes. J. Food Eng..

[26] Fellows, P. J. Extrusion cooking. in *Food Processing Technology: Principles and Practice* (ed. Fellows, P. J.). 753–780. (Elsevier, 2017).

[27] Ayed, C. *et al.* The role of sodium chloride in the sensory and physico-chemical properties of sweet biscuits. *Food Chem. X* **9**, 100115 (2021). 10.1016/j.fochx.2021.100115 PMC7817490 33511340

